# Effects of Phytocannabinoids on Reproductive System and Prenatal Development: Mechanisms and Clinical Implications

**DOI:** 10.3390/jcm14186494

**Published:** 2025-09-15

**Authors:** Michał Wesołowski, Aleksandra Sobaś, Kamil Biedka, Jakub Karwacki, Jakub Bulski, Katarzyna Błaszczyk, Kacper Żełabowski, Oliwia Ziobro, Filip Jacek Maj, Karol Sornat, Agata Estreicher, Anna Klasa, Andrzej Dłubak, Tadeusz Sebzda

**Affiliations:** 1 Department of Physiology and Pathophysiology, Division of Pathophysiology, Wroclaw Medical University, 10 Chalubinskiego Street, 50-368 Wroclaw, Poland; aleksandra.ewasobas@gmail.com (A.S.); kamil.biedka@umw.edu.pl (K.B.); jkarwacki.md@gmail.com (J.K.); blaszczykrzyk@gmail.com (K.B.); oziobro@gmail.com (O.Z.); agataestreicher@gmail.com (A.E.); tadeusz.sebzda@umw.edu.pl (T.S.); 2 Faculty of Medicine, Collegium Medicum, Jan Kochanowski University, 19A IX Wiekow Kielc Avenue, 25-317 Kielce, Poland; jakub.bulski@icloud.com (J.B.); filip.jacek.maj@gmail.com (F.J.M.); karol.b.sornat@gmail.com (K.S.); 3 University Center of Excellence in Urology, Department of Minimally Invasive and Robotic Urology, Wroclaw Medical University, 213 Borowska Street, 50-556 Wroclaw, Poland; 4 Scientific Society for Psychopharmacology, Department of Forensic Medicine, Wroclaw Medical University, 4 J. Mikulicza-Radeckiego Street, 50-345 Wroclaw, Poland; kacper.zelabowski@outlook.com; 5 University Hospital in Kraków, 2 Jakubowskiego Street, 30-688 Kraków, Poland; klasa.annamaria@gmail.com; 6 Department of Internal Medicine, District General Hospital, 1 Baczynskiego Street, 55-200 Oława, Poland

**Keywords:** cannabis, endocannabinoid system, THC, marijuana, reproduction, spermatogenesis, pregnancy, prenatal, postnatal

## Abstract

Cannabis is one of the most studied psychoactive substances due to its increasing prevalence and evolving legal status. Of particular concern is the rising consumption among young individuals, where excessive use may disrupt reproductive processes and pose long-term health risks to offspring. This narrative review examines the effects of cannabis use on male and female reproductive health, including its impact on male fertility, the female reproductive system, placental function, and prenatal and postnatal outcomes, as well as fetal development. A nonsystematic review was conducted using PubMed, Scopus, Web of Science, and Google Scholar databases in November 2024. After screening titles and abstracts and the full-text analysis, 64 studies were included in this narrative review. In men, cannabinoids can interfere with spermatogenesis, reduce sperm motility and quality, and lower testosterone levels, as demonstrated in clinical and experimental studies. In women, cannabinoid-induced disorders include negative effects on ovarian follicle maturation, ovulation, placental function, and prenatal development. Prenatal exposure to cannabis is associated with the risk of reduced birth weight, birth defects, sudden infant death syndrome (SIDS) or lactation problems due to the penetration of cannabis metabolites into breast milk. The findings highlight the potential negative effects of cannabis on reproductive health and fetal development. Given these risks, individuals attempting to conceive, and pregnant women should be advised against cannabis use. Greater awareness is needed among healthcare professionals and the public regarding the reproductive risks associated with cannabis consumption. While the evidence on teratogenic effects is not always conclusive, caution should be exercised, and further research is essential to deepen the understanding of these effects.

## 1. Introduction

Cannabis is one of the most widely used psychoactive drugs in the world, with use increasing rapidly since the 1960s, particularly among young people. The legalization of cannabis in countries such as Canada, Thailand, Uruguay, Malta, Mexico, Georgia and South Africa have been associated with an increase in its use. Estimates suggest that 209 million people worldwide have had experience with cannabis use. Recent studies have indicated an increase in both the frequency of use and the number of regular users [1]. In the National Cannabis Survey (NCS), the percentage of people using cannabis in the past three months increased from 14% in 2018 to 20% in 2020. The highest prevalence of cannabis use was observed among the 18–44 age group, which increased from 28% in 2018 to 36% in 2020. Similarly, in the 25–44 age group, the prevalence of cannabis use reached 30% in 2020, marking a significant increase from 21% in 2018. A similar trend was observed among individuals over 45, with a rise from 7% in 2018 to 11% in 2020, indicating a 4 percentage point increase. [2]. Recent data (2022–2024) confirm that cannabis use continues to rise [3]. In the United States, the 2022 National Survey on Drug Use and Health reported that 22–25% of the population aged ≥12 years had used cannabis in the past year, and 15% in the past month [4]. Young adults (19–30 years) show particularly high rates, with 42% reporting past-year use, 29% past-month use, and 10% using daily or near-daily [5]. Among university students, a 30-year increase in past-month cannabis use has been observed (26.1% in 2023 vs. 16.1% in 1988) [6]. In Australia, the 2023–2024 National Drug Strategy Household Survey found that 41% of individuals aged ≥ 14 years had tried cannabis at least once in their lifetime, and 11.5% had used it in the past year [7].

According to a study by F. Navarrete et al., the prevalence of cannabis use among pregnant women aged 15–44 was found to be over 4.9%, rising to 8.5% in the 18–25 age bracket [8]. It has been demonstrated that cannabis use during pregnancy is more prevalent among younger women, those with lower socioeconomic status, and individuals with limited access to prenatal care [9]. Prenatal cannabis exposure is particularly harmful during the first trimester, which represents the most critical period of organogenesis. Younger women are at increased risk of unintentional exposure, as pregnancy is often unrecognized at this stage. Furthermore, younger women more frequently report cannabis use for both recreational purposes and for the alleviation of anxiety or nausea, which further elevates the likelihood of early gestational exposure [10].

Cannabinoid is a chemical compound that is found naturally in the body, in the cannabis plant, or produced synthetically. Accordingly, the distinction is made between phytocannabinoids, endocannabinoids, and synthetic cannabinoids. Phytocannabinoids are present in plants such as *Cannabis sativa* and *Cannabis indica*. Endocannabinoids are produced in the body and act as lipophilic neurotransmitters via cannabinoid receptors. Cannabis contains more than 400 different chemicals, including more than 60 cannabinoid compounds. The main psychoactive cannabinoid in cannabis is Δ9-tetrahydrocannabinol (THC), which interacts with the endocannabinoid system [11].

This narrative review aimed to examine the effects of phytocannabinoids on the male and female reproductive systems and their impact on prenatal development. The study highlights mechanisms through which cannabis influences reproductive health and discusses the clinical implications of its use, particularly in the context of pregnancy.

## 2. Methods

This review was prepared as a narrative synthesis of available evidence on the effects of phytocannabinoids on reproductive health. In November 2024, a structured literature search was conducted in PubMed, Scopus, Web of Science, and Google Scholar. The search strategy included both MeSH/Emtree terms and free-text keywords related to cannabis and reproductive processes, such as “cannabis,” “marijuana,” “tetrahydrocannabinol,” “cannabidiol,” “endocannabinoid system,” “reproduction,” “fertility,” “placenta,” “pregnancy,” “offspring,” “prenatal,” and “postnatal.”

We included original research articles and systematic reviews that assessed the effects of phytocannabinoids on reproductive health or prenatal/postnatal development. Eligible study designs encompassed human observational studies, animal models, in vitro experiments, and hybrid translational studies. Narrative commentaries, editorials, conference abstracts without complete data, and studies unrelated to reproductive outcomes were excluded.

Given the considerable heterogeneity of study designs, populations, exposures, and outcome measures, no quantitative meta-analysis was performed. Instead, findings were synthesized qualitatively. Formal tools for risk-of-bias or quality assessment were not applied, consistent with the narrative character of this review.

## 3. Endocannabinoid System

**Figure 1:**
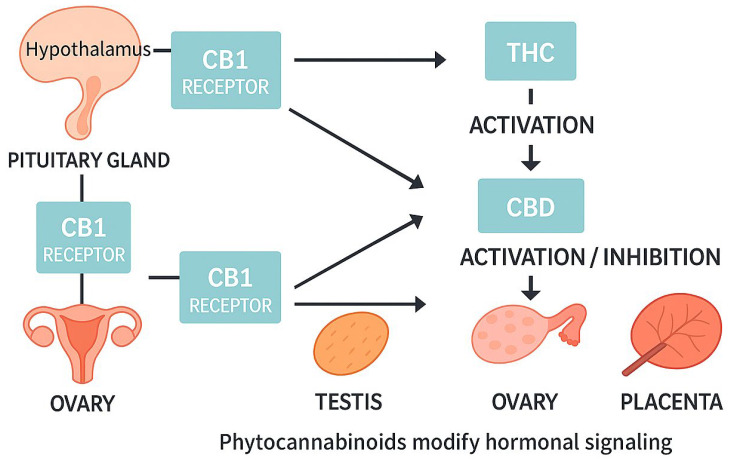
Schematic overview of cannabinoid receptor type 1 distribution in the reproductive neuroendocrine axis and peripheral organs (hypothalamus, pituitary gland, ovary, testis, placenta), and the modulatory role of phytocannabinoids (Δ9-tetrahydrocannabinol, cannabidiol) on hormonal signaling pathways. THC—Δ9-tetrahydrocannabinol, CBD—cannabidiol.

The endocannabinoid system (ECS) comprises a series of interconnected components, including endogenous cannabinoids, cannabinoid receptors, and the enzymes responsible for their synthesis and degradation. These receptors are distributed across both the central and peripheral nervous systems [12]. In addition to their fundamental functions, cannabinoid receptors act as targets for THC, a psychoactive substance present in cannabis. These receptors are distributed throughout the body, with the most well-known, cannabinoid receptor type 1 (CB1) and type 2 (CB2), found in both the central and peripheral nervous systems. CB1 receptors are localized in the hypothalamus and pituitary gland, which are key components of the reproductive neuroendocrine axis, as well as in peripheral organs such as the ovary, testis, and placenta. (Figure 1). Endogenous cannabinoids play a key role in regulating many physiological and cognitive processes [13,14]. The two main compounds found in cannabis are THC and cannabidiol (CBD). THC acts as a partial agonist at CB1 and CB2 receptors, showing high affinity for CB1, which is responsible for its psychoactive effects [15]. CBD, a non-psychoactive phytocannabinoid found in cannabis, has a complex, multidirectional mechanism of action. It has been demonstrated to influence a variety of physiological systems, including the cannabinoid, serotonergic, and opioid systems. However, its affinity for CB1 and CB2 receptors is comparatively low. Within the cannabinoid system, its mechanism of action may involve negative allosteric modulation [16].

## 4. Phytocannabinoids and the Male Reproductive System

The mechanisms controlling male reproductive health include endocrine, paracrine, and autocrine regulation. These mechanisms are strongly linked to environmental and lifestyle factors. The endocannabinoid system plays a critical role in reproduction. The CB1 receptor has been identified in testes and sperm, and the presence of receptors and their ligands has also been confirmed in seminal vesicles and corpora cavernosa [17,18].

ECS constitutes a critical regulatory network governing reproductive physiology through its modulation of the hypothalamic–pituitary–gonadal (HPG) axis. Cannabinoid receptor type 1 (CB1), expressed within the hypothalamus and pituitary gland, exerts control over the pulsatile release of gonadotropin-releasing hormone (GnRH). This, in turn, modulates the secretion of luteinizing hormone (LH) and follicle-stimulating hormone (FSH), thereby influencing gonadal steroidogenesis [9,19]. Engagement of CB1 receptors by THC initiates Gi/o protein–coupled signaling cascades that suppress cyclic adenosine monophosphate/protein kinase A (cAMP/PKA) activity. This inhibition downregulates the synthesis of neuropeptides essential for reproductive endocrine regulation [19,20].

Within gonadal tissues, including Leydig cells in the testes and granulosa cells in the ovaries, activation of CB1 and CB2 receptors disrupts steroid biosynthesis. This occurs, in part, through the suppression of cytochrome P450 enzymes, such as aromatase, leading to altered production of estrogens, progesterone, and testosterone [10,20].

ECS plays a pivotal role in the regulation of male reproduction, and its disruption can have deleterious effects on various stages of germ cell development. As previously mentioned, disturbances in ECS function have been shown to affect gametogenesis, sperm motility, and sperm capacitation [21].

Emerging evidence suggests that certain phytocannabinoids, particularly CBD and CBG, may exert anti-cancer effects on prostate cancer (PCa) cells through mechanisms such as mitochondrial dysfunction, metabolic reprogramming, and apoptosis induction [22]. These compounds influence mitochondrial bioenergetics via interactions with voltage-dependent anion channel 1 (VDAC1) and hexokinase II (HKII), while also modulating oncogenic signaling pathways, including the PI3K/Akt/mTOR axis [22,23]. Inhibition of these pathways reduces PCa cell viability and enhances apoptosis, suggesting potential adjuvant applications. Importantly, cannabinoids also act through CB1 and CB2 receptors, which are expressed in reproductive tissues, highlighting mechanistic links between the endocannabinoid system, oncogenesis, and reproductive endocrinology. However, emerging data indicate a possible dual role of cannabinoid signaling, with context-dependent effects that may promote as well as inhibit cancer progression [23]. Research indicates that CBD increases glycolytic activity while inhibiting oxidative phosphorylation in enzalutamide-resistant hormone-resistant PCa cells [21]. While these findings are noteworthy, the clinical relevance remains uncertain, and further studies are required to clarify implications for male reproductive health.

### 4.1. Reduction in Sperm Concentration and Total Sperm Count

A study conducted by Gundersen et al. indicates a negative effect of cannabis use on semen quality. The study’s participants were 1215 men aged 18 to 28 from the Danish population who were participating in qualifying for military service. The study’s findings revealed that individuals who consumed cannabis more than once a week exhibited a significant decline in sperm concentration, with a 28% reduction, and a substantial decrease in the total number of sperm, with a 29% decrease, when compared to individuals who did not use cannabis. Furthermore, a positive correlation was identified between the frequency of cannabis use and a decline in sperm count [24].

In a separate study, the impact of CBD administration on testicular weight was examined using an animal model involving rhesus monkeys [25]. The results demonstrated that CBD administration led to a significant reduction in testicular weight, ranging from 20% to 25%. Following the cessation of CBD administration and a 30-day recovery period, testicular weight did not return to baseline values. A reduction in spermatogenesis was also observed, which showed a correlation with the CBD dose administered. Higher doses were associated with a greater decrease in sperm production in the tested individuals.

Conversely, Murphy et al. conducted a study on a group of 24 men, half of whom used cannabis and the other half of whom did not. The study revealed that men who used cannabis had lower sperm levels compared to the control group [26].

### 4.2. Variations in Sperm Structure and DNA

Sperm morphology refers to the sperm’s shape and structure, which are crucial to their ability to fertilize an egg. Abnormal sperm morphology has been demonstrated to have a deleterious effect on sperm motility and fertilization potential [27]. Research has identified anomalies in sperm morphology in individuals who use marijuana, as evidenced by studies examining male fertility [28].

Mitochondria, the primary source of energy for spermatozoa, play a pivotal role in ensuring their motility. The CB1 receptor, a key player in this process, is found not only in the sperm head but also within the mitochondrial membrane within the cell. Cannabinoids have been shown to inhibit oxygen consumption in the mitochondria, which may result in impaired sperm motility by disrupting their energy processes [29].

Research in both human cohorts and controlled animal models has demonstrated that cannabis use affects the epigenetic profile of sperm. Rodent studies have shown that cannabinoid exposure alters DNA methylation patterns in germ cells, with consequences for genes involved in early development. Correspondingly, analyses in human sperm samples from cannabis users have revealed differential methylation of developmentally relevant genes such as DLGAP2 [17]. Another example is the PTGIR gene, which encodes the prostacyclin receptor (PGI2) and regulates sperm motility. Hypomethylation of PTGIR has been observed in sperm from cannabis users and has been associated with reduced reproductive capacity. These findings highlight how both preclinical models and human data consistently link cannabinoid exposure with epigenetic remodeling of sperm [26].

### 4.3. Effects on Endocrine Metabolism in Men

**Figure 2:**
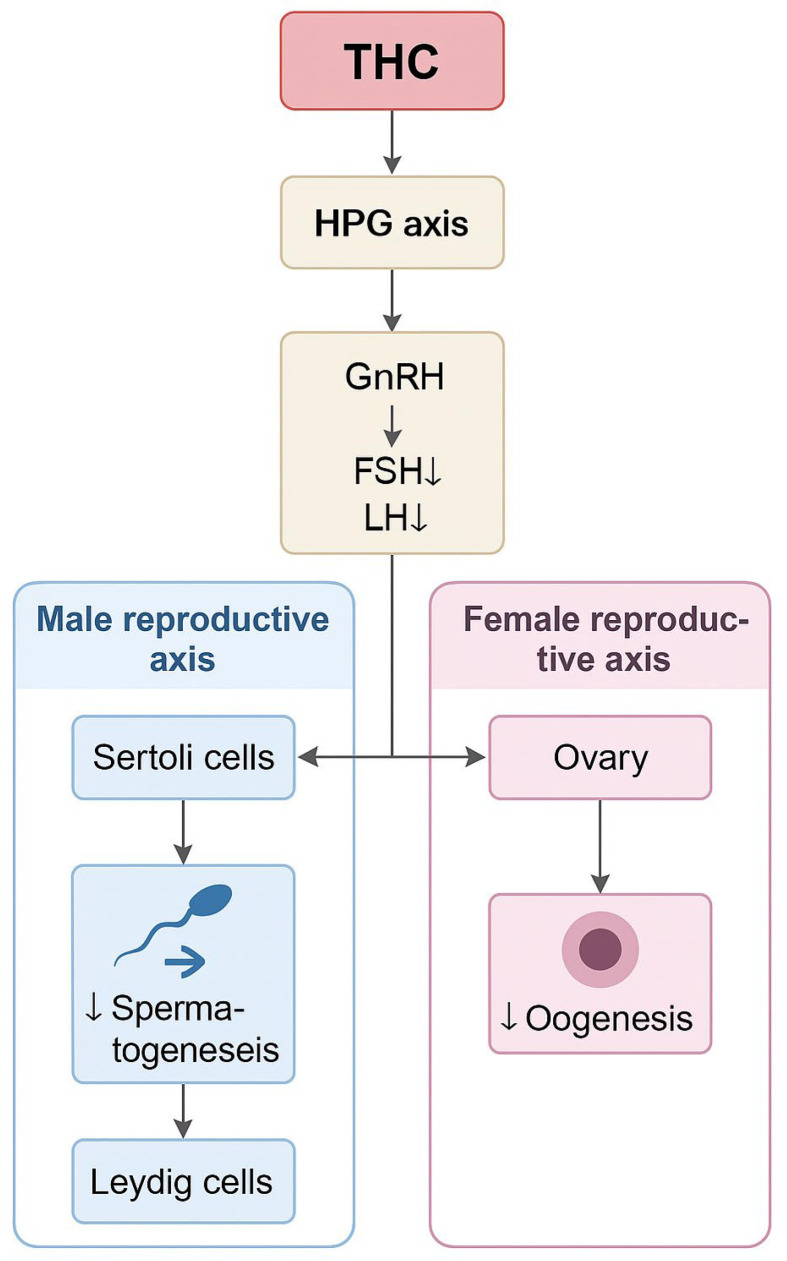
Schematic representation of the mechanisms by which tetrahydrocannabinol (THC) disrupts the hypothalamic-pituitary-gonadal (HPG) axis, leading to altered levels of gonadotropin-releasing hormone (GnRH), follicle-stimulating hormone (FSH), and luteinizing hormone (LH). These changes impair the function of Sertoli and Leydig cells in males—leading to reduced spermatogenesis—and disrupt follicular development and oogenesis in females. The diagram delineates the male and female reproductive axes in response to THC exposure. ↓—decrease.

Spermatogenesis, the process of sperm cell formation, requires the proper functioning of the hypothalamic–pituitary–gonadal axis. This involves the release of GnRH from the hypothalamus, which stimulates the secretion of follicle-stimulating hormone (FSH) and luteinizing hormone (LH) from the pituitary gland. Follitropin has been found to promote spermatogenesis by affecting Sertoli cells, while lutropin activates Leydig cells in the testes to synthesize testosterone. The presence of endocannabinoid receptors in both the hypothalamus and pituitary gland has been confirmed, indicating a role for the endocannabinoid system in the regulation of these processes. However, cannabis competing for receptor sites has been shown to disrupt hormone feedback mechanisms by interfering with normal spermatogenesis. The presence of CB1 receptors in Leydig cells has been demonstrated, and the use of cannabis has been associated with a decrease in the activity of esterase isoenzymes present in these cells, thereby reducing testosterone synthesis [25,30,31,32]. These hormonal mechanisms, as well as the parallel effects on female reproductive function and oogenesis, are summarized in Figure 2.

## 5. Phytocannabinoids and the Female Reproductive System

The presence of receptors belonging to the endocannabinoid system has been identified in various tissues and organs within the female reproductive system. These include the ovaries, fallopian tubes, uterus, and placenta. Specifically, CB1 and CB2 receptors have been detected in ovarian cells, including Graaf follicle cells. These receptors play a crucial role in various aspects of the female reproductive system, such as follicle maturation, ovulation, and hormonal regulation.

ECS plays a major role in various reproductive processes, including the transport of the embryo through the fallopian tube, the development of the placenta, and embryo implantation. The ECS has been shown to interact with the hypothalamic-pituitary-ovarian axis, exhibiting intricate links to the production and release of steroid hormones. The ECS’s multidirectional effects underscore its important role in regulating reproductive mechanisms. The active compounds present in cannabis have been shown to exert substantial effects on the female reproductive system, exerting a detrimental impact on reproductive health and fertility-related processes [33,34].

### Ovulation and Hormonal Management

The ovaries are responsible for producing female gametes and synthesizing estrogen and progesterone. The ovary’s primary functional unit is the follicle, which envelops the oocyte, supplying it with vital nutrients and molecular signals, while ensuring its protection [11]. The maturation of both the follicle and the oocyte is controlled by gonadotropic hormones, FSH and LH, whose secretion is regulated by the hypothalamic-pituitary-ovarian axis. Research has demonstrated that exogenous cannabinoids disrupt the balance of this hormonal axis, exerting a detrimental effect on processes associated with germ cell maturation [35].

Cannabinoid-related interference in hypothalamic function is well documented, with evidence showing that these compounds can disturb the maturation cycle of the Graafian follicle as well as the ovulatory process. A study by Lammert and colleagues evaluated women using marijuana and tobacco concurrently compared to women using tobacco alone. The findings indicated that the group using marijuana and tobacco had a shorter luteal phase compared to those who used tobacco exclusively. Lammert et al. examined women who co-used marijuana and tobacco, matching them by age to a tobacco-only group to partially account for age-related menstrual cycle differences. While this design allowed some separation of marijuana’s effects from those of tobacco, co-exposure complicates attributing luteal-phase changes solely to cannabis. Phase lengths were measured consistently using LH tests, but the small sample size and lack of detailed information on marijuana dose and frequency limit the ability to fully adjust for confounding factors, which should be considered when interpreting the results. [36]. In animal models, THC has been demonstrated to exert a direct inhibitory effect on FSH-regulated processes, which are indispensable for steroid biosynthesis and the induction of LH receptor expression, both essential for ovarian follicle maturation [37]. Chronic exposure to exogenous cannabinoids has been demonstrated to exert adverse effects on ovarian follicle development [38]. A comparative overview of the effects of phytocannabinoids on male and female reproductive physiology is presented in Table 1.

## 6. Phytocannabinoids’ Effect on the Placenta and Prenatal Development

The placenta, a transient organ connecting the fetus to the maternal body via the umbilical cord, plays a pivotal role in fetal development. It facilitates the transfer of oxygen and nutrients, removes metabolites from the fetal circulation, and provides protection against harmful agents such as maternal pathogens, xenobiotics, environmental toxins, and certain drugs. This protective role is mediated through multiple mechanisms, including the physical barrier formed by the syncytiotrophoblast, enzymatic detoxification systems, and the activity of placental transporters that limit the passage of potentially harmful compounds. Abnormal placental formation can adversely impact fetal and future postnatal development [41]. The use of cannabis during pregnancy has been associated with potential risks to fetal development. Studies have demonstrated that cannabinoids can cross the placental barrier, and THC exposure has been associated with placental failure [42,43].

Additionally, THC exposure has been associated with impaired angiogenesis and reduced oxygen delivery to the fetus, which may contribute to adverse effects on nervous system development [39,44]. In an in vitro model using the HTR-8/SVneo cell line, cannabinoids were shown to disrupt tube formation, indicating angiogenesis dysregulation [40]. The neuronal system of the embryo exhibits heightened sensitivity to exposure resulting from maternal cannabis abuse, which can result in abnormalities in the development of behavioral, cognitive, and neuropsychiatric functions [45].

Depending on the duration of THC exposure, it has been observed that it can affect the expression of genes related to metabolic enzymes in the placenta [39]. Furthermore, exposure to cannabis during pregnancy has been observed to induce endoplasmic reticulum stress, resulting in premature cell cycle arrest in placental tissue [46]. A study by Nassan et al. found that marijuana use correlated with an increased risk of miscarriage [47].

Furthermore, there is a possibility that cannabis use during pregnancy may be associated with serious abnormalities, such as intrauterine fetal growth restriction (IUGR) and preeclampsia [48]. A study by Abdelwahab et al. demonstrated that exposure to cannabis was associated with an elevated risk of small-of-gestational age (SGA) below the 10th percentile. The incidence of SGA among cannabinoid users was found to be 35%, which was higher than the rate observed for use of other substances [49]. Furthermore, newborns whose mothers used cannabis during pregnancy were more likely to require hospitalization in an intensive care unit [39]. Reinforcing the evidence, a cohort study by Metz et al. demonstrated that maternal cannabis use, confirmed by urinary biomarkers, is associated with an increased risk of adverse pregnancy outcomes linked to placental dysfunction. Continuous exposure beyond the first trimester increased the risk of a composite primary outcome, including SGA birth, preterm delivery, stillbirth, and gestational hypertension (adjusted RR 1.32; 95% CI 1.09–1.60) [50]. A summary of the key prenatal and postnatal consequences of cannabis exposure, categorized by affected system, is provided in Table 2.

The prevailing belief that cannabis, a naturally derived substance, can alleviate vomiting symptoms in pregnant women is also supported by this evidence. However, the potential risks associated with its use significantly outweigh the possible benefits, making the use of antiemetic drugs with a documented safety profile a more appropriate alternative [52].

A study by Crume et al. identified younger age, low educational attainment, and lower socioeconomic status as significant factors contributing to increased cannabis use during pregnancy [55].

## 7. Newborn and Breastfeeding

It has been demonstrated that exposure to cannabis during the prenatal period can lead to a multitude of serious consequences that will significantly affect the development and functioning of the child after birth. A mounting body of evidence has associated prenatal exposure to cannabis with an elevated risk of developmental anomalies, including congenital malformations within the nervous system. These anomalies may include microcephaly, premature fusion of cranial sutures, brainlessness, and neural tube defects [51].

Furthermore, newborns exposed to marijuana during the prenatal period demonstrate an elevated risk of congenital defects within the urinary and renal systems. The most prevalent anomalies include renal agenesis, hydronephrosis, polycystic kidney disease, and the presence of a congenital posterior urethral valve (PUV) [56].

Prenatal exposure to cannabis has been linked to the manifestation of withdrawal syndrome symptoms in newborns. Furthermore, neonatal exposure to cannabis has been associated with an elevated risk of sudden infant death syndrome (SIDS) [52].

Lactation is a natural physiological process initiated in a woman’s body with the conception of a child. The components of breast milk play a pivotal role in the normal development of the newborn [57]. The presence of cannabis metabolites, including THC, in breast milk has been observed to vary significantly depending on the dosage of the substance consumed by the lactating mother. Research indicates that THC levels in breast milk can reach levels up to eight times higher than those found in the blood when cannabis is regularly used during breastfeeding [38]. Despite the paucity of knowledge regarding the specific effects of cannabis exposure during the neonatal and infant period, results from preclinical lactation studies suggest potential effects on neural network development [58]. Maternal exposure to cannabis has been shown to affect maternal nutrition, leading to an involuntary reduction in food and water intake. This, in turn, results in a supply of essential nutrients to the fetus that may not meet its needs. Inadequate maternal diet during the prenatal period, as well as after birth, has been associated with abnormalities in the development of the newborn [59].

## 8. Prenatal Phytocannabinoid Exposure and Long-Term Effects on Offspring Development

The effects of THC, a principal psychoactive component of cannabis, on neural development have been a subject of considerable research interest. Studies have demonstrated that THC exerts a significant influence on interneuronal communication processes and the maturation of the nervous system. Prenatal exposure to THC has been associated with alterations in the structural and functional integrity of the nervous system [60]. Moreover, research has indicated that exposure to cannabis during pregnancy has been associated with developmental delays in infants during their initial years of life [61].

Furthermore, cannabis use has been associated with the development of neurological disorders that emerge during pregnancy and persist throughout life. These disorders have been observed as associations with deficits in short-term memory, impulse control, and attention problems [51]. Children who are prenatally exposed to cannabis demonstrate challenges in spatial skills, as well as in speech and math skills, in comparison to peers who were not prenatally exposed to cannabis. Postnatal irritability, as well as an increased tendency towards aggressive behavior and criminal activities during early adolescence, have been associated with prenatal cannabis exposure. These phenomena may be partly explained by the effects of psychoactive substances present in cannabis on the development of the prefrontal cortex and other brain structures involved in regulating emotion, memory, and cognitive function [62,63].

**Figure 3:**
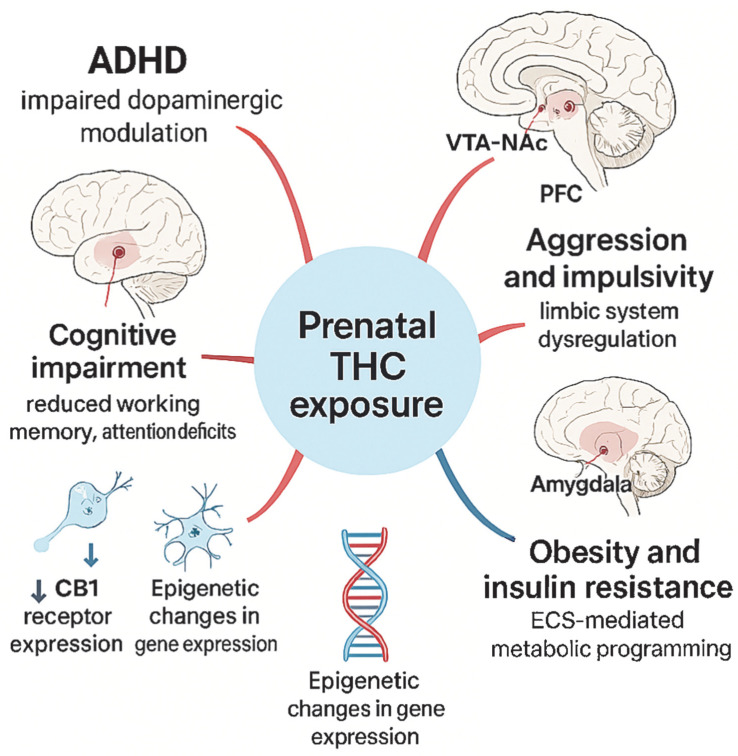
Long-term neurobehavioral and metabolic consequences of prenatal THC exposure. Prenatal exposure to THC affects central nervous system development through altered dopaminergic modulation, limbic system dysregulation, and metabolic programming. Affected brain structures include the prefrontal cortex (PFC), hippocampus, amygdala, and mesolimbic dopaminergic pathway (VTA–NAc). Molecular mechanisms include CB1 receptor downregulation and epigenetic changes in gene expression, leading to increased risk of ADHD, cognitive impairment, aggression, and metabolic disorders such as obesity and insulin resistance. ↓—decrease.

Furthermore, exposure to cannabis during pregnancy has been associated with an elevated risk of depression, anxiety disorders, and disruptions to bedtime rhythms, including sleep problems, in children exposed to the substance [54]. Prenatal cannabis exposure can lead to several serious health effects on offspring, including metabolic disorders. Prenatal exposure to cannabis has been linked to an increased risk of obesity and elevated fasting glucose levels in childhood in children whose mothers used it during pregnancy [55,64]. Figure 3 illustrates the proposed mechanisms linking prenatal THC exposure with long-term neurobehavioral and metabolic effects.

## 9. Future Directions and Perspectives

Given the increasing prevalence of phytocannabinoid use among individuals of reproductive age, further research in this area is essential. Future studies should focus on several key directions. Large-scale, prospective, and long-term human studies are needed to better define the associations between cannabis exposure and reproductive or developmental outcomes, while accounting for potential confounding factors such as tobacco and alcohol co-use, socioeconomic status, and maternal nutrition. At the same time, molecular and cellular studies are required to elucidate the pathways through which phytocannabinoids influence the hypothalamic–pituitary–gonadal axis, gametogenesis, and placental function. Particular attention should be paid to epigenetic alterations in germ cells, which may result in long-term and even transgenerational consequences. Another important avenue for research is the evaluation of dose–response relationships, the identification of critical windows of vulnerability during pregnancy, and the differentiation of effects related to specific cannabinoids and modes of consumption, including the risks associated with passive exposure, for example, among pregnant women. It must be emphasized that direct interventional studies in pregnant women are severely constrained by ethical considerations and given the potential harmful effects of cannabis on the fetus, such research remains especially difficult to conduct. Therefore, well-designed observational studies, animal models, and translational approaches will continue to play a central role in advancing knowledge in this field. From a clinical and public health perspective, future strategies should include the development of evidence-based guidelines for healthcare professionals, the implementation of routine screening for cannabis use during preconception and prenatal care, and the establishment of educational programs aimed at raising awareness of potential reproductive and developmental risks.

### Limitations

This review has several limitations that should be acknowledged. The measurement of cannabis exposure varied across studies, with many relying on self-reported use, which is prone to recall bias and underreporting, whereas others used biomarkers (e.g., urine or blood THC levels), providing more objective but less frequently available data. Cannabis potency and formulations have changed substantially over recent decades, with contemporary products often containing higher concentrations of Δ9-THC and novel preparations (e.g., edibles, concentrates, vaping liquids), which complicates comparisons across studies conducted in different time periods. The timing of exposure was heterogeneous: while some studies specifically evaluated first-trimester exposure, others assessed continued use throughout pregnancy, and relatively few addressed dose–response relationships or critical windows of vulnerability. Finally, there is a scarcity of longitudinal human studies extending into late childhood and adolescence; therefore, many of the long-term neurobehavioral and metabolic outcomes remain uncertain and are primarily inferred from animal models or short-term follow-up. These limitations highlight the need for cautious interpretation and underscore the importance of future well-designed, prospective studies.

## 10. Conclusions

The sociocultural acceptance of cannabis legalization has the potential to engender the misconception that it is harmless, a notion that is sometimes associated with its natural origin. However, the results of scientific studies clearly indicate the negative effects of cannabis constituents on prenatal and postnatal development of offspring. Children exposed to cannabinoids during pregnancy are more likely to be born with reduced birth weight and require hospitalization in neonatal intensive care units. In addition, there is an increased risk of cognitive, neuropsychiatric, and metabolic disorders in later life. Marijuana exposure can also negatively affect the functioning of endocrine and reproductive systems in men and women of childbearing age. Considering the current scientific evidence, it is necessary to conduct extensive educational activities targeting people of reproductive age, especially pregnant women, to raise awareness about the potential risks of marijuana use and its harmful effects on fetal development. Given the expanding prevalence of cannabis use among reproductive-age individuals, there is an urgent need for further research to elucidate the underlying mechanisms of cannabis use and to formulate precise, evidence-based guidelines that can inform decisions regarding cannabis use, not only in pregnant women but also in the reproductive population seeking to conceive.

## Figures and Tables

**Table 1:** Summary of the effects of phytocannabinoids on male and female reproductive systems.

System/Process	Observed Effect	Evidence Type	References
Hormonal disruption	↓ Testosterone, altered HPG axis	Human, animal	[23,24,25,29,30,31]
Gametogenesis	↓ Spermatogenesis, sperm motility, and viability	Human, animal	[23,24,25,29]
Structural anomalies	Abnormal sperm morphology; altered DNA methylation (DLGAP2, PTGIR)	Human, animal	[17,26,32]
Cancer-related impact	Modulation of prostate cancer growth via CB1/CB2	In vitro, animal	[21,22,23]
Female hormonal regulation	Disrupted HPG axis, ↓ FSH/LH	Human, animal	[33,34,35]
Oogenesis/folliculogenesis	Impaired follicular maturation, ovulation; shortened luteal phase	Human, animal	[34,35,39]
Endocannabinoid expression	CB1/CB2 in testes, sperm, Leydig cells; CB1/CB2 in ovaries, uterus, placenta	Human, animal	[9,36,40]

HPG—hypothalamic–pituitary–gonadal axis; FSH—follicle-stimulating hormone; LH—luteinizing hormone; DLGAP2—Discs Large Homolog-Associated Protein 2; PTGIR—Prostaglandin I2 Receptor; CB1/CB2—cannabinoid receptors type 1 and 2; ↓—decrease.

**Table 2:** Adverse effects of prenatal cannabis exposure on pregnancy, placenta, fetus, and offspring.

System/Stage	Observed Effect (with Citation)	Mechanism/Directionality	Evidence Type
Pregnancy	↑ Risk of preterm birth, preeclampsia, miscarriage [47,48,51]	Impaired trophoblast invasion, altered cytokines	Human cohorts
Placental function	↓ Angiogenesis, ↓ VEGF, oxidative, and ER stress [40,41,42,43,46]	Mitochondrial dysfunction, inflammation, apoptosis	In vitro, animal
Fetal development	Intrauterine growth restriction (IUGR), low birth weight, SGA < 10th percentile [49,50]	Impaired oxygen/nutrient transport, placental insufficiency	Human cohorts
Neonatal outcome	↑ NICU admission, ↑ stillbirth risk [44,50]	Placental dysfunction	Human cohorts
Neurodevelopment (child)	Cognitive impairment, ADHD, emotional dysregulation, ↑ SIDS risk [46,52]	Altered endocannabinoid signaling in hippocampus/PFC	Human, animal
Long-term metabolic risk	↑ Obesity, ↑ fasting glucose in childhood [53,54]	Epigenetic reprogramming, insulin resistance pathways	Human cohorts

VEGF—vascular endothelial growth factor; ER—endoplasmic reticulum; IUGR—intrauterine growth restriction; SGA—small-for-gestational age; NICU—neonatal intensive care unit; ADHD—attention-deficit/hyperactivity disorder; SIDS—sudden infant death syndrome; PFC—prefrontal cortex; ↑—increase; ↓—decrease.

## Data Availability

Not applicable.

## Abbreviations

The following abbreviations are used in this manuscript:

CBD	cannabidiol
CBG	cannabigerol
CB1	cannabinoid receptor type 1
CB2	cannabinoid receptor type 2
ECS	endocannabinoid system
FSH	follicle-stimulating hormone
GnRH	gonadotropin-releasing hormone
HKII	hexokinase II
HPG axis	hypothalamic–pituitary–gonadal axis
IUGR	intrauterine growth restriction
LH	luteinizing hormone
mTOR	mammalian target of rapamycin
NCS	national cannabis survey
PCa	prostate cancer
PGI2	prostacyclin receptor
PI3K	phosphatidylinositol-3-kinase
PUV	posterior urethral valve
SGA	small-of-gestational age
SIDS	sudden infant death syndrome
THC	δ9-tetrahydrocannabinol
VDAC1	voltage-dependent anion channel 1

## References

[1] Matheson J., Le Foll B. (2023). Impacts of Recreational Cannabis Legalization on Use and Harms: A Narrative Review of Sex/Gender Differences. Front. Psychiatry.

[2] Hall W., Stjepanović D., Dawson D., Leung J. (2023). The Implementation and Public Health Impacts of Cannabis Legalization in Canada: A Systematic Review. Addiction.

[3] Baldwin G.T., Whitehill J.M., Levinson A.H., Kerridge B.T., Keyes K.M. (2024). Current Cannabis Use in the United States, 2002–2022. Am. J. Public Health.

[4] Substance Abuse Mental Health Services Administration (SAMHSA) (2023). 2022 National Survey on Drug Use and Health: Detailed Tables.

[5] National Institute on Drug Abuse (NIDA) (2024). Cannabis and Hallucinogen Use Among Adults Remained at Historic Highs in 2023. News Release. https://nida.nih.gov/news-events/news-releases/2024/08/cannabis-and-hallucinogen-use-among-adults-remained-at-historic-highs-in-2023.

[6] Drug Abuse Statistics College Marijuana Use Statistics 2023. https://drugabusestatistics.org.

[7] Australian Institute of Health and Welfare (AIHW) (2025). Alcohol, Tobacco & Other Drugs in Australia: Cannabis. https://www.aihw.gov.au/reports/alcohol/alcohol-tobacco-other-drugs-australia/contents/drug-types/cannabis.

[8] Navarrete F., García-Gutiérrez M.S., Gasparyan A., Austrich-Olivares A., Femenía T., Manzanares J. (2020). Cannabis Use in Pregnant and Breastfeeding Women: Behavioral and Neurobiological Consequences. Front. Psychiatry.

[9] Young-Wolff K.C., Adams S.R., Alexeeff S.E., Zhu Y., Chojolan E., Slama N.E., Does M.B., Silver L.D., Ansley D., Castellanos C.L. (2024). Prenatal Cannabis Use and Maternal Pregnancy Outcomes. J. Clin. Med..

[10] Baía I., Domingues R. (2024). The Effects of Cannabis Use During Pregnancy on Low Birth Weight and Preterm Birth: A Systematic Review and Meta-Analysis. Am. J. Perinatol..

[11] Czarnywojtek A., Borowska M., Dyrka K., Moskal J., Kościński J., Krela-Kaźmierczak I., Lewandowska A.M., Abou Hjeily B., Gut P., Hoffmann K. (2023). The Influence of Various Endocrine Disruptors on the Reproductive System. Endokrynol. Pol..

[12] Matei D., Trofin D., Iordan D.A., Onu I., Condurache I., Ionite C., Buculei I. (2023). The Endocannabinoid System and Physical Exercise. Int. J. Mol. Sci..

[13] Hill M.N., Haney M., Hillard C.J., Karhson D.S., Vecchiarelli H.A. (2023). The Endocannabinoid System as a Putative Target for the Development of Novel Drugs for the Treatment of Psychiatric Illnesses. Psychol. Med..

[14] Lu H.-C., Mackie K. (2021). Review of the Endocannabinoid System. Biol. Psychiatry Cogn. Neurosci. Neuroimaging.

[15] Garani R., Watts J.J., Mizrahi R. (2021). Endocannabinoid System in Psychotic and Mood Disorders, a Review of Human Studies. Prog. Neuro-Psychopharmacol. Biol. Psychiatry.

[16] Capodice J.L., Kaplan S.A. (2021). The Endocannabinoid System, Cannabis, and Cannabidiol: Implications in Urology and Men’s Health. Curr. Urol..

[17] Mazzeo F., Meccariello R. (2023). Cannabis and Paternal Epigenetic Inheritance. Int. J. Environ. Res. Public Health.

[18] Srinivasan M., Hamouda R.K., Ambedkar B., Arzoun H.I., Sahib I., Fondeur J., Escudero Mendez L., Mohammed L. (2021). The Effect of Marijuana on the Incidence and Evolution of Male Infertility: A Systematic Review. Cureus.

[19] Brik M., Sandonis M., Hernández-Fleury A., Gil J., Mota M., Barranco F.J., Garcia I., Maiz N., Carreras E. (2024). Cannabis exposure during pregnancy and perinatal outcomes: A cohort study. Acta Obstet. Gynecol. Scand..

[20] Lo J.O., Shaw B., Robalino S., Ayers C.K., Durbin S., Rushkin M.C., Olyaei A., Kansagara D., Harrod C.S. (2024). Cannabis Use in Pregnancy and Neonatal Outcomes: A Systematic Review and Meta-Analysis. Cannabis Cannabinoid Res..

[21] Mahmoud A.M., Kostrzewa M., Marolda V., Cerasuolo M., Maccarinelli F., Coltrini D., Rezzola S., Giacomini A., Mollica M.P., Motta A. (2023). Cannabidiol Alters Mitochondrial Bioenergetics via VDAC1 and Triggers Cell Death in Hormone-Refractory Prostate Cancer. Pharmacol. Res..

[22] Lal S., Shekher A., Puneet, Narula A.S., Abrahamse H., Gupta S.C. (2021). Cannabis and Its Constituents for Cancer: History, Biogenesis, Chemistry and Pharmacological Activities. Pharmacol. Res..

[23] Acharya B., Sahu P.K., Behera A., Feehan J., Mishra D.P., Apostolopoulos V. (2025). Cannabinoids and the Male Reproductive System: Implications of Endocannabinoid Signaling Pathways. Maturitas.

[24] Gundersen T.D., Jørgensen N., Andersson A.-M., Bang A.K., Nordkap L., Skakkebæk N.E., Priskorn L., Juul A., Jensen T.K. (2015). Association Between Use of Marijuana and Male Reproductive Hormones and Semen Quality: A Study Among 1215 Healthy Young Men. Am. J. Epidemiol..

[25] Gingrich J., Choudhuri S., Cournoyer P., Downey J., Jacobs K.M. (2023). Review of the Oral Toxicity of Cannabidiol (CBD). Food Chem. Toxicol..

[26] Murphy S.K., Itchon-Ramos N., Visco Z., Huang Z., Grenier C., Schrott R., Acharya K., Boudreau M.-H., Price T.M., Raburn D.J. (2018). Cannabinoid Exposure and Altered DNA Methylation in Rat and Human Sperm. Epigenetics.

[27] Pelzman D.L., Sandlow J.I. (2024). Sperm Morphology: Evaluating Its Clinical Relevance in Contemporary Fertility Practice. Reprod. Med. Biol..

[28] Pacey A.A., Povey A.C., Clyma J.-A., McNamee R., Moore H.D., Baillie H., Cherry N.M., Participating Centres of Chaps-UK (2014). Modifiable and Non-Modifiable Risk Factors for Poor Sperm Morphology. Hum. Reprod..

[29] Barchi M., Innocenzi E., Giannattasio T., Dolci S., Rossi P., Grimaldi P. (2019). Cannabinoid Receptors Signaling in the Development, Epigenetics, and Tumours of Male Germ Cells. Int. J. Mol. Sci..

[30] Gammon C.M., Freeman G.M., Xie W., Petersen S.L., Wetsel W.C. (2005). Regulation of Gonadotropin-Releasing Hormone Secretion by Cannabinoids. Endocrinology.

[31] Carvalho R.K., Andersen M.L., Mazaro-Costa R. (2020). The Effects of Cannabidiol on Male Reproductive System: A Literature Review. J. Appl. Toxicol..

[32] Lo J.O., Hedges J.C., Girardi G. (2022). Impact of Cannabinoids on Pregnancy, Reproductive Health, and Offspring Outcomes. Am. J. Obstet. Gynecol..

[33] Martínez-Peña A.A., Lee K., Pereira M., Ayyash A., Petrik J.J., Hardy D.B., Holloway A.C. (2022). Prenatal Exposure to Delta-9-Tetrahydrocannabinol (THC) Alters the Expression of miR-122-5p and Its Target Igf1r in the Adult Rat Ovary. Int. J. Mol. Sci..

[34] Walker O.S., Holloway A.C., Raha S. (2019). The Role of the Endocannabinoid System in Female Reproductive Tissues. J. Ovarian Res..

[35] Fonseca B.M., Rebelo I. (2022). Cannabis and Cannabinoids in Reproduction and Fertility: Where We Stand. Reprod. Sci..

[36] Lammert S., Harrison K., Tosun N., Allen S. (2018). Menstrual Cycle in Women Who Co-Use Marijuana and Tobacco. J. Addict. Med..

[37] Randhawa J., Smith A.L., Patel N., Chen Y., Garcia M., Martinez L. (2025). Characterizing the Role of Endocannabinoid Receptor Cnr1 in FSH-Mediated Steroidogenesis and LH Receptor Expression in Rat Granulosa Cells. Cell Biochem. Funct..

[38] Cecconi S., Rapino C., Di Nisio V., Rossi G., Maccarrone M. (2020). The (Endo) Cannabinoid Signaling in Female Reproduction: What Are the Latest Advances?. Prog. Lipid Res..

[39] Hayer S., Mandelbaum A.D., Watch L., Ryan K.S., Hedges M.A., Manuzak J.A., Easley C.A., Schust D.J., Lo J.O. (2023). Cannabis and Pregnancy: A Review. Obstet. Gynecol. Surv..

[40] Alves P., Amaral C., Gonçalves M.S., Teixeira N., Correia-da-Silva G. (2024). Cannabidivarin and cannabigerol induce unfolded protein response and angiogenesis dysregulation in placental trophoblast HTR-8/SVneo cells. Arch. Toxicol..

[41] Rokeby A.C.E., Natale B.V., Natale D.R.C. (2023). Cannabinoids and the Placenta: Receptors, Signaling and Outcomes. Placenta.

[42] Lojpur T., Easton Z., Raez-Villanueva S., Laviolette S., Holloway A.C., Hardy D.B. (2019). Δ9-Tetrahydrocannabinol Leads to Endoplasmic Reticulum Stress and Mitochondrial Dysfunction in Human BeWo Trophoblasts. Reprod. Toxicol..

[43] Ritchie T.M., Feng E., Vahedi F., Ermolina S., Bellissimo C.J., De Jong E., Portillo A.L., Poznanski S.M., Chan L., Ettehadieh S.M. (2025). The impact of oral cannabis consumption during pregnancy on maternal spiral artery remodelling, fetal growth and offspring behaviour in mice. eBioMedicine.

[44] Brar B.K., Patil P.S., Jackson D.N., Gardner M.O., Alexander J.M., Doyle N.M. (2021). Effect of Intrauterine Marijuana Exposure on Fetal Growth Patterns and Placental Vascular Resistance. J. Matern. Fetal Neonatal Med..

[45] Vargish G.A., Pelkey K.A., Yuan X., Chittajallu R., Collins D., Fang C., McBain C.J. (2017). Persistent Inhibitory Circuit Defects and Disrupted Social Behaviour Following in Utero Exogenous Cannabinoid Exposure. Mol. Psychiatry.

[46] Ezechukwu H.C., Diya C.A., Shrestha N., Hryciw D.H. (2020). Role for Endocannabinoids in Early Pregnancy: Recent Advances and the Effects of Cannabis Use. Am. J. Physiol.-Endocrinol. Metab..

[47] Nassan F.L., Arvizu M., Mínguez-Alarcón L., Gaskins A.J., Williams P.L., Petrozza J.C., Hauser R., Chavarro J.E. (2019). EARTH Study Team Marijuana Smoking and Outcomes of Infertility Treatment with Assisted Reproductive Technologies. Hum. Reprod..

[48] Huppertz B. (2018). The Critical Role of Abnormal Trophoblast Development in the Etiology of Preeclampsia. Curr. Pharm. Biotechnol..

[49] Abdelwahab M., Klebanoff M.A., Venkatesh K.K. (2022). Association Between Prenatal Marijuana and Tobacco Smoke Exposures and Small for Gestational Age at Birth. Am. J. Perinatol..

[50] Metz T.D., Allshouse A.A., McMillin G.A., Greene T., Chung J.H., Grobman W.A., Haas D.M., Mercer B.M., Parry S., Reddy U.M. (2023). Cannabis Exposure and Adverse Pregnancy Outcomes Related to Placental Function. JAMA.

[51] Lin A., Dent G.L., Davies S., Dominguez Z.M., Cioffredi L.-A., McLemore G.L., Maxwell J.R. (2023). Prenatal Cannabinoid Exposure: Why Expecting Individuals Should Take a Pregnancy Pause from Using Cannabinoid Products. Front. Pediatr..

[52] Badowski S., Smith G. (2020). Cannabis Use During Pregnancy and Postpartum. Can. Fam. Physician Med. Fam. Can..

[53] Nashed M.G., Hardy D.B., Laviolette S.R. (2021). Prenatal Cannabinoid Exposure: Emerging Evidence of Physiological and Neuropsychiatric Abnormalities. Front. Psychiatry.

[54] Moore B.F., Sauder K.A., Shapiro A.L.B., Crume T., Kinney G.L., Dabelea D. (2022). Fetal Exposure to Cannabis and Childhood Metabolic Outcomes: The Healthy Start Study. J. Clin. Endocrinol. Metab..

[55] Crume T.L., Juhl A.L., Brooks-Russell A., Hall K.E., Wymore E., Borgelt L.M. (2018). Cannabis Use During the Perinatal Period in a State with Legalized Recreational and Medical Marijuana: The Association Between Maternal Characteristics, Breastfeeding Patterns, and Neonatal Outcomes. J. Pediatr..

[56] Reece A., Hulse G. (2022). Epidemiological Patterns of Cannabis- and Substance- Related Congenital Uronephrological Anomalies in Europe: Geospatiotemporal and Causal Inferential Study. Int. J. Environ. Res. Public Health.

[57] Jabłońska A. (2022). Laktacja—Rola Karmienia Piersią i Jej Wpływ Na Rozwój Dziecka. Innow. Pielęgniarstwie Nauk. Zdrowiu.

[58] Bara A., Ferland J.-M.N., Rompala G., Szutorisz H., Hurd Y.L. (2021). Cannabis and Synaptic Reprogramming of the Developing Brain. Nat. Rev. Neurosci..

[59] Mulligan M.K., Hamre K.M. (2023). Influence of Prenatal Cannabinoid Exposure on Early Development and Beyond. Adv. Drug Alcohol Res..

[60] Grant K.S., Petroff R., Isoherranen N., Stella N., Burbacher T.M. (2018). Cannabis Use during Pregnancy: Pharmacokinetics and Effects on Child Development. Pharmacol. Ther..

[61] Martin G.I. (2020). Marijuana: The Effects on Pregnancy, the Fetus, and the Newborn. J. Perinatol..

[62] Murnan A.W., Keim S.A., Yeates K.O., Boone K.M., Sheppard K.W., Klebanoff M.A. (2021). Behavioral and Cognitive Differences in Early Childhood Related to Prenatal Marijuana Exposure. J. Appl. Dev. Psychol..

[63] Baranger D.A.A., Paul S.E., Colbert S.M.C., Karcher N.R., Johnson E.C., Hatoum A.S., Bogdan R. (2022). Association of Mental Health Burden with Prenatal Cannabis Exposure from Childhood to Early Adolescence: Longitudinal Findings from the Adolescent Brain Cognitive Development (ABCD) Study. JAMA Pediatr..

[64] Moore B.F. (2024). Prenatal Exposure to Cannabis: Effects on Childhood Obesity and Cardiometabolic Health. Curr. Obes. Rep..

